# Does the backlift also matter in female cricket? A biomechanical investigation among local and international female cricket batters

**DOI:** 10.17159/2078-516X/2025/v37i1a22315

**Published:** 2025-10-15

**Authors:** A Patel, H Noorbhai

**Affiliations:** BEAHT Research Centre, Faculty of Health Sciences, University of Johannesburg

**Keywords:** women’s cricket, cricket batting, biomechanics, performance

## Abstract

**Background:**

Despite extensive research on batting biomechanics among male cricketers, limited studies focus on female cricket players.

**Objectives:**

This study examines the biomechanical and performance implications of the lateral batting backlift technique (LBBT) and straight batting backlift technique (SBBT) among female cricketers.

**Methods:**

This study aims to bridge this gap by assessing the backlift, its influence on shot execution and its relationship with performance metrics among local (n=18; South African National Women’s Cricket Team, also known as the Protea (P) Women’s Cricket team (n=9) and non-Protea players (NP) n=9), as well as international (n=34) female players, using high-speed video analysis and StanceBeam bat sensors. Statistical analyses, including Spearman’s Rho correlations and Mann-Whitney U tests, were applied to evaluate key performance metrics such as bat speed, impact speed, and wagon wheel shot distribution.

**Results:**

Findings show that local (67%) and international (62%) players used the LBBT, which was associated with greater performance metrics, e.g., strong positive correlations were found between bat speed and impact speed (r=0.85, p<0.001). A significant difference in wagon wheel shot distribution was observed between P and NP players (p=0.039).

**Conclusion:**

Findings suggest that the LBBT offers biomechanical advantages and may be a key contributing factor in optimising female batting performance. Future longitudinal research studies should focus on three-dimensional (3D) motion capture (as well as markerless motion capture) on the LBBT, integrating real-time analytics and expanding sample sizes to include diverse player populations.

Cricket batting techniques have received substantial attention due to their significant impact on a team’s ability to score runs.^[1]^ A critical component of a batter’s skill set is their batting backlift technique (BBT). This influences a batter’s ability to generate power, time their shots and adapt to different bowling styles. The BBT is typically classified into two distinct types: the straight batting backlift technique (SBBT) and the lateral batting backlift technique (LBBT).^[2]^ While the LBBT has been extensively studied among male cricketers and linked to higher performance outcomes, research on its effectiveness among female players remains limited.^[3,4]^

Biomechanical studies have provided valuable insights into batting techniques through the use of high-speed video analysis and motion-tracking technology.^[5]^ These tools allow researchers to examine key performance indicators such as bat speed, impact force and shot placement, offering a deeper understanding of how technique influences performance outcomes.^[6,7]^ However, most existing studies focus on male players, leaving a significant gap in knowledge regarding female batting biomechanics. Given the physiological and biomechanical differences between male and female athletes, it is crucial to determine how these distinctions affect the execution and success of the BBT in female cricket.^[8]^

Few studies have consistently highlighted differences in batting biomechanics between male and female cricketers.^[9]^ Male players typically exhibit greater muscle mass, increased bat speed and higher ball launch speeds, which contribute to longer shot carry distances.^[6]^ Conversely, female players rely more on lower-body mechanics and hip drive to compensate for differences in upper-body strength. These distinctions influence the effectiveness and execution of the BBT, particularly in power-hitting scenarios.^[10]^

While previous work has explored the mechanical advantages male players derive from employing the LBBT (such as enhanced bat arc trajectories and improved kinetic chain coordination), comparable investigations in female cricket remain limited. Female batters likely adapt their technique differently, relying on greater lower-limb impulse and rotational hip torque to generate power.^[6]^ These adaptations may have important implications for coaching and conditioning strategies aimed at optimising performance among female athletes.

The LBBT has been associated with greater shot adaptability and power generation, allowing batters to access a broader range of scoring areas on the field.^[2]^ Male cricketers employing the LBBT have demonstrated higher batting averages and scoring efficiency, yet similar trends in female cricket remain underexplored. Given the rising prominence of women’s T20 and ODI formats, understanding how BBT influences shot selection and scoring patterns in female cricket is essential for enhancing player development and performance strategies.^[11]^

The biomechanical analysis of cricket batting involves evaluating kinematic parameters such as bat speed, impact speed, follow-through angle, backlift angle and downswing angles.^[5]^ Studies have shown that male players generate higher bat acceleration and momentum transfer, which contributes to greater power-hitting efficiency.^[9]^ Female players tend to compensate by utilising hip rotation, stance adjustments and a more controlled backlift to generate shot power.^[6,12]^

Coaching strategies in cricket have historically been developed with male players in mind, often overlooking the physiological and biomechanical differences that affect female players’ performance.^[8,13]^

Coaching programmes should emphasise lower-body strength development, hip rotation efficiency and grip adjustments to optimise BBT effectiveness among female players.^[6]^ The results of this study will contribute to a more gender-inclusive approach to cricket coaching, ensuring that female athletes receive tailored training regimens that align with their biomechanical capabilities and performance.^[14]^

This study aimed to enhance understanding of BBT among professional female cricketers by analysing its prevalence, biomechanical effectiveness, and impact on performance through comprehensive video and biomechanical analyses. It also strives to bridge gender gaps in cricket biomechanics by examining the influence of gender-specific factors on BBT.

## Methods

### Study design

This study utilised a cross-sectional design employing analytical research methods to investigate the BBT used by professional female batters, in accordance with the STROBE guidelines for observational studies.^[15]^ This design facilitated the collection and analysis of numerical and statistical data from a pre-determined sample of female batters to discover trends and averages.

### Sample selection

The sample selection took place in two parts. The first was the local player (LP) selection and recruitment, and the second was the international player (IP) selection, where the data were extracted from online sources. This study combines international players’ match video analysis with local biomechanical testing to fully investigate the use and implications of the two types of BBT. The local data offers detailed performance metrics, while the international footage contextualises these findings within elite match-play scenarios.

#### Local player selection

The study included 18 participants (local players = LP) (n=18), all professional female cricket players. This sample comprised uninjured, healthy, professional players who were 18 years or older from the Protea [South African National Women’s Cricket Team] (P) (n=9) and non-Protea players (NP) (n=9).

#### International sample selection

Additional online sample selection included the top 34 international female batters (IP), with a career batting average higher than 30.00 in Women’s One Day Internationals (WODIs) as detailed by ESPN Cricinfo, and who played at least 25 matches.

### Ethical considerations

Ethical clearance was obtained from the Faculty of Health Sciences Research Ethics Committee (REC-1764-2022) at the University of Johannesburg, South Africa. The international player data analysed in this study were sourced from publicly accessible YouTube videos and ESPN Cricinfo statistics. As per standard practice in sports science research involving publicly available data, no specific permissions were required for the reuse of these materials. Only publicly available match footage was used, and no personally identifiable or sensitive information was collected or reported beyond what is already in the public domain. This approach aligns with ethical research practices involving open-source data.

### Data collection

#### Procedure

This study followed a two-phase data collection procedure to address sample size limitations and enhance the robustness of the findings. Initially, the study aimed to assess batting backlift techniques using biomechanical data collected from local professional female cricket players through direct testing. Due to the limited sample size in this group, a second phase of data collection was implemented using publicly available video footage of international players to supplement and contextualise local findings.

In Phase 1, biomechanical and anthropometric data were collected from local players in a controlled training environment. This included high-speed video analysis and StanceBeam Smart Bat Sensor data collection. Participants underwent standardised measurements and hit 12 balls using their gear.

In Phase 2, video footage of international female cricketers was sourced based on the predefined selection criteria, ensuring footage quality, camera angle consistency and relevance for BBT classification. This additional dataset enabled comparative analysis of technique distribution across local and international cohorts and provided a broader context for the biomechanical findings (Table 1).

#### Local testing

##### Height

The height measure (HM200P) Portable Stadiometer was used to measure the participants’ height (Charder Medical, Taiwan) to the nearest millimetre (mm), with participants standing upright, barefoot, and in light clothing.

##### Weight

The Omron Kadra Scan Body Composition Monitor and Scale (201809-00264F) was used to measure the participant’s weight.

##### Waist circumference

Waist circumference was measured using a standard Fizique BMI measuring tape. A horizontal measurement was taken at the narrowest point on the torso, above the umbilicus and below the xiphoid process, while the participant was standing straight, with their feet together, arms at their sides and their abdomen relaxed.

##### Body mass index

Body mass index was calculated using the players’ body mass in relation to their height using the formula:

BMI=body mass (kg)/height (m^2^).

The body mass index is used in this study as a metric for preliminary anthropometric analysis to provide a broad depiction of players’ physical composition. However, BMI has disadvantages, particularly for professional athletes, whose greater muscular mass might lead to exaggerated readings that don’t fairly represent body fat or athletic ability.

Although BMI and waist circumference are traditionally used in general health assessments, their inclusion in this study contributed to a comprehensive anthropometric profile of professional female cricketers. While these variables are not direct indicators of performance, they were examined in relation to batting metrics such as bat speed and wagon wheel distribution to explore potential associations between body composition and biomechanical output. The inclusion of body fat percentage alongside BMI and waist circumference allowed for a more nuanced understanding of physical morphology within a performance-based context.

This study employed the American College of Sports Medicine (ACSM)^[15]^ guidelines, which were used to measure the anthropometric variables such as body fat percentage, waist circumference and BMI.

##### Body fat percentage

Body fat percentage was measured using the Fizique Slim Guide Skinfold Calipers. The Montreal Olympic Games Anthropometric Project (MOGAP) formula uses the sum of the six skinfold sites in millimetres (mm). The six skinfold sites are the triceps brachii, subscapularis, abdomen, suprailliac, thigh and calf. The gender-specific MOGAP formula for females calculated the body fat percentage, as follows: Body fat percentage = [(∑ 6 skinfolds × 0.1548) + 3.58].

All skinfolds were taken on the right side of the body in accordance with ACSM guidelines.^[16]^ All measurements were recorded to the nearest millimetre (mm), directly on the skin beneath light clothing where applicable, ensuring accuracy and consistency across participants.

##### Biomechanical testing

To perform biomechanical testing, participants had to complete a warm-up of 6–10 minutes (at a light intensity: Borg RPE of 9–11) before testing. Participants wore their own protective gear and used their own cricket bats. This approach ensured ecological validity by replicating real match conditions and maintaining the individual comfort and familiarity associated with their standard equipment. The use of each participant’s own bat was particularly important for capturing natural movement patterns during batting, as subtle changes in bat type can influence grip, swing mechanics and timing. Similarly, the use of their regular protective gear allowed players to perform in a manner consistent with competitive play, without introducing discomfort or restrictions that could alter biomechanical outputs.

##### StanceBeam cricket bat sensor

The Smart Cricket Bat Sensor-SSTD-1912-01934 (by StanceBeam Performance Analytics Solutions) was connected to the StanceBeam iOS Mobile Application (Quicklogi Technologies India Pvt Ltd) via Bluetooth. The pitch length was subsequently entered via the application, and the Striker Sensor was positioned away from the wickets. The sensor was attached to each participant’s bat handle at the proximal end using a bat mount to keep the sensor striker in place before participants went to bat. This was used to monitor real-time sessions in which the backlift angles, downswing/follow-through angles, wagon wheels, bat speeds, impact speeds and maximum power were recorded. At the end of the session, a summary was produced of all the variables collected (Supplementary Figures 1 and 2).

##### Video and data capture

A high-speed video camera (JVC GC-PX100, measuring 250–255 frames per second) was positioned 1.5m behind the pitch. The tripod was set up at 1.1 m height, facing the wickets, so that the batter and the bowler’s delivery arm could be seen in the frame. Each batter positioned themselves in front of the wickets within the camera frame. Subsequently, they faced 12 balls (two overs). Video footage captured both the delivery and the batting performance. A right-arm bowler was used to deliver the balls to the players, rather than a bowling machine. This was to replicate real match conditions better. Bowling machines were initially considered but dismissed due to their lack of variability and realism. All bowlers delivered from a consistent length and style to minimise variability. This setup was selected to maintain ecological validity in biomechanical testing. The footage captured both the key aspects of the delivery and the batting performance. Specifically, the photos used for further analysis depicted the exact moment the ball left the batter’s hand. The StanceBeam cricket bat sensor captured the batter’s variables and produced an online session report (Supplementary Figures 1, 2 and 3). In addition, an inter-rater technique was utilised to reduce bias and perspective error for camera calibration.

#### International video data collection

##### Video data capture

Publicly available video footage from local and international cricket matches was utilised to analyse the players’ BBTs. These videos were sourced from YouTube, which focused on public match highlights or game-day footage, with recent matches having the best video quality. Each player had six videos selected for analysis, with specific criteria ensuring consistency and relevance. The selected videos had to depict the wickets, the batter facing the camera, and the moment the ball left the bowler’s hand. Only footage of right-arm bowlers (right-arm fast, right-arm medium, right-arm medium-fast, right-arm off-break) was used to maintain consistency in the analysis, ensuring that variations in bowling style did not confound the analysis of the backlift technique. The bowler’s identity and bowling style were confirmed beforehand to align with the video criteria.

### Data analysis

#### Anthropometry and field data of local players

The hard copy data sheets from the clinical tests were recorded onto a Microsoft Excel spreadsheet, along with participant information, their cricket teams and anthropometry. Overall averages from StanceBeam, namely: bat speed (km·h^−1^), impact speed (km·h^−1^), time to impact (milliseconds), downswing angle [degrees], follow-through angle [degrees], number of wagon wheels (on-side, off-side and straight); as well as the maximum bat speed (km·h^−1^) and maximum impact speed (km·h^−1^). Vertical and horizontal shots were split and recorded into sub-categories of backlift heights in degrees, as well as classifications of high, medium, horizontal shots were split and recorded into sub-categories of backlift heights in degrees, as well as classifications of high, medium and low (Supplementary Figure 3).

#### Local video data

Video data from the cameras was collected and evaluated using the Kinovea analysis system software. Kinovea (Kinovea-0.9.5; Kinovea Open-Source Project) - a two-dimensional (2D) motion-capture analysis system, which measured the kinematic parameters by utilising the chronometer to measure lengths of time, as well as the line, joint angles and goniometer instruments to gauge distances and backlift angles of the participants. These variables were calculated using the batter’s performance during the phase analysis of their batting performance. The phases of significant areas of movement were the preliminary phase (backlift), retraction (swing execution), action (ball impact) and follow-through.^[17]^ The relative (joint) angles of interest were the degree of extension between the thigh and shank segments, front knee extension, stride length, wrist cocking angle and elbow flexion/extension. These classifiers were coded as follows: Classifier A represented the bat’s toe pointing straight back or facing the first slip (between 0° and 25°); Classifier B indicated the bat’s toe pointing between the second and third slips (between 25° and 45°); Classifier C denoted the bat’s toe pointing towards the gully as well as if the bat’s face pointing towards the off-side (between 45° and 80°). The classification of the open-faced backlift was based on the direction of the bat’s toe, regardless of the angle it formed, if the bat’s face was open. The classifiers were split into Classifier A as the SBBT and Classifiers B and C as the LBBT (Supplementary Figure 4).

#### International video data

Precise lift angles may not be accurately determined for the video footage of the additional international batters; therefore, angle ranges were used to determine the classifiers. Analysing the backlift at various positions and periods during the lift enhanced the validity and reliability of the analysis.^[18]^ To determine the direction of the bat’s toe, a standard method involved drawing three lines and vectors: a vertical line (yellow line) from the head to the hands, a horizontal line (red line) indicating the hand position and an oblique line (blue line) representing the orientation of the bat during the backlift.^[2]^ The still image captured from the video footage, taken from the last frame before the ball’s release, was analysed. These lines formed an angle illustrating the bat’s distance from the body in the frontal plane and the amount of rotation before ball contact. To account for perspective errors, the researchers restricted the analysis to specific footage with different deliveries (off-side, middle and leg-side; full length, back of a length and short length) and incorporated horizontal lines in the backdrop. The results were tabulated with the player’s wagon wheel data (Supplementary Figure 5).

These performance metrics collected are crucial because they provide objective, quantitative data on a player’s batting technique. Analysing these variables provides a better understanding of how a player’s technique affects their on-field performance. Furthermore, comparing these measurements to morphological data allows investigation of how physical qualities, i.e. body composition may influence or improve batting performance.

### Statistical analysis

The data from the participants were analysed using inferential statistical analysis, which aided in developing inferences regarding the study population. As the sample size was less than 50 people, the Shapiro-Wilk test determined the data’s normality. Parametric and non-parametric tests were used when applicable, depending on the distribution of the data. Spearman’s correlation coefficient was used to establish statistically significant correlations between the selected morphological, biomechanical and performance metrics. The strength and direction of these correlations were determined by interpreting the R statistic value (r), which is positive for a direct relationship and negative for an inverse relationship between the variables. The SPSS Statistics software programme (Version 26, IBM, USA) was used for statistical analysis to evaluate the study’s results. All statistical tests were performed at a significance level of p<0.05.

## Results

### Local players

The selected morphological data included height, weight, BMI, waist circumference and body fat percentage. The average BMI was slightly above the normal range (25.2±3.1), while waist circumference remained within healthy limits (<80 cm). The MOGAP skinfold formula estimated body fat percentage at 22.5±3.7%.

The results showed that the LBBT was the dominant technique among local players (67%). LBBT players showed slightly higher bat speed (37.8 km·h^−1^) than SBBT players (35.8 km·h^−1^), but the difference was not statistically significant. Impact speed and downswing angle followed similar trends, with no significant differences. Wagon wheel shot distribution was the only significant difference, with LBBT players favouring on-side shots (p=0.039). BMI, waist circumference and body fat percentage were positively correlated (r>0.7, p<0.05), confirming their interdependence. Bat speed correlated strongly with impact speed and downswing angle (r>0.8, p<0.05). Wagon wheel on-side shots were negatively correlated with BMI, suggesting that higher BMI may influence shot selection.

Players were grouped into Protea (P) and Non-Protea (NP) categories. No significant differences in BMI, waist circumference or body fat percentage (p>0.05) were noted. Wagon wheel shot selection varied significantly (p=0.039), with Protea players favouring on-side shots.

Table 3 shows the Mann-Whitney U test findings of the number of participants (N) and the mean, standard deviation and mean rank for each group. The mean rank is essential since each group’s relative positions for the data values are indicated in this table. Interpreting the mean and standard deviation in non-parametric environments was performed cautiously, even if they provide a general idea of each group’s variability and central tendency.^[19]^ The P groups’ mean ranks are compared to those of the NP groupings.

Table 2 observed no statistically significant difference between BMI, waist circumference and skinfolds. When comparing BMI, waist circumference and skinfolds between P and NP, there is no statistically significant difference between the groups for these variables.

#### Spearman’s Rho correlation analysis

Correlation analysis between performance metrics showed that there were strong positive correlations between bat speed, impact speed, downswing angle and follow-through angle (r>0.8, p<0.05). A negative correlation between BMI and wagon wheel on-side shots indicated a potential link between body composition and shot selection (Supplementary Table 1). However, due to the number of correlations, results should be interpreted with caution as there is an increased risk of Type I error.

### International players

**Fig. 1:**
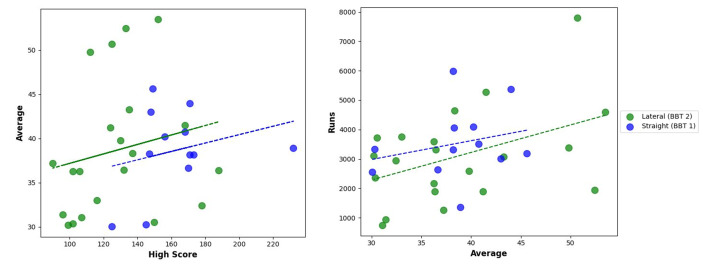
High scores versus averages and averages versus runs of international players (lateral vs straight)

Video analysis of international players (n=34) was categorised into LBBT (62%) and SBBT (38%) groups. The LBBT players scored more runs and had higher high scores, but the difference was not statistically significant. Scatter plots showed a weak positive correlation between batting averages and total runs for both groups. The LBBT players had a wider spread of high scores and runs, suggesting greater adaptability in shot execution (Figure 1).

#### Cross-tabulations between local and international players

The distribution of BBT among local and international players was not statistically significant (p=0.771). Both groups showed a preference for LBBT over SBBT.

Table 3 displays the Mann-Whitney U test results for the number of participants and their mean, standard deviation and mean rank for each group. The distribution of BBT among local and international cricket players was studied using cross-tabulation analysis.

Table 4 shows that 67 % (n=12) of the 18 local players preferred the LBBT, while 33 % (n=6) used the SBBT. On the other hand, of the 34 international players, 38 % (n=13) used the SBBT and 62 % (n=21) the LBBT. Overall, 37 per cent of players in both groups used the SBBT, with 64 % used the LBBT. A Fisher’s Exact Test was used to examine whether the type of BBT (local vs international) had a statistically significant relationship. The Fisher’s Exact Test returned a p-value of 0.771. The null hypothesis cannot be rejected because the p-value exceeds the conventional alpha criterion (0.05). This suggests that the distribution of BBT between local and international players is not statistically significant.

## Discussion

The study’s findings highlight the significance of certain BBTs in improving performance outcomes, particularly among female players. These are congruent with the original premise and closely coincide with previous studies on male cricketers’ use of their specific BBT. Among both the participants and the local video data (n=52), a significant majority (63.5%) adopted the LBBT, demonstrating its widespread use among professional female cricketers. The evidence supports that the LBBT also assists male players and benefits female cricketers considerably. This consistency shows that the biomechanical concepts behind the LBBT may benefit both males and females. The results of this study suggest that the LBBT may offer biomechanical advantages in power generation and shot adaptability. Coaches of female cricket players could incorporate LBBT-focused drills, including hip rotation exercises, timing drills, and grip adjustments. Training programmes might benefit from placing greater emphasis on lower-body mechanics to compensate for upper-body strength differences.

### Batting technique studies of male and female cricket batters

Previous research on the BBT of male cricket players at the amateur and elite levels supports these findings, and 70% of the all-time greatest batters adopted the LBBT.^[2,10,20]^ However, those research papers differed regarding gender focus, bigger sample sizes and the number of kinematic variables examined. Notably, the prior studies on female cricket batting techniques^[6,7,12]^ did not include precise batting data obtained from the StanceBeam Bat Sensor (markerless motion capture), which has proven critical in providing fresh insights to the literature. These measures have made it possible to conduct a more thorough examination and gain greater knowledge of how the LBBT influences effective batting. This enhanced ability to associate the LBBT with batting statistics verifies the technique’s benefits.

### Anthropometry

The analysis of the BBT among professional female cricket players revealed no statistically significant differences between the groups in terms of BMI, waist circumference or skinfold measurements. With all p-values exceeding the 0.05 significance level, the results indicate that anthropometric factors do not appear to play a decisive role in the adoption or effectiveness of either the SBBT or the LBBT. While this study did not find significant anthropometric differences among BBT users, it highlights the need for further research with larger sample sizes and a broader range of performance variables. Previously, there has been a positive correlation between a batter’s lean body mass and their ability to deliver strong strikes in cricket. The batter’s maximum muscle strength aids in increasing the beginning velocity of the bat swing, allowing the batter to reach the ball speed for a successful and powerful stroke.^[21]^ Despite the absence of clear anthropometric distinctions, the study provides valuable insights into the prevalence and distribution of BBT among female players. This supports the idea that the LBBT may offer biomechanical advantages that enhance batting performance, regardless of body composition.

### Performance metrics

Additionally, performance metrics gathered from the StanceBeam Bat Sensor showed certain trends in bat speed, impact speed and downswing angles, which may have practical implications for coaching strategies. However, the non-significant statistical differences suggest that further research is necessary to establish conclusive relationships between BBT and performance metrics. Further, results identified a positive correlation between maximum bat speed and maximum impact speed. However, a previous study using the StanceBeam smart bat sensor identified no differences in maximum bat speed and maximum impact speed.^[22]^

The wagon wheel data indicated a considerable tendency towards on-side shots, with fewer shots going straight and off-side. While using the LBBT, which may be most suited for bringing the ball to the on-side, previous research suggests the LBBT was superior at scoring runs in various areas of the cricket field.^[10]^ The highest measured data, including impact and peak bat speeds, indicate the LBBT’s capacity to produce high-velocity strokes. It is important to note that the wagon wheel data had a limited sample size, which would have influenced the results. The few metrics that were acquired, particularly of the straight and off-side, may have created bias or reduced the validity of the patterns discovered. This constraint suggests that, while the data provides useful insights into the players’ behaviours and as a practising aid for game scenarios, caution should be exercised when drawing from these findings.^[18,23]^

### Local players versus international players

Lastly, cross-tabulation between local and international players also indicated no statistically significant differences in BBT adoption. This finding suggests that coaching practices rather than developmental factors may play a more substantial role in shaping batting technique. Future studies should explore additional biomechanical and tactical aspects of batting techniques to provide more definitive conclusions about the factors influencing BBT adoption and effectiveness for both males and females.

### Limitations

One of the key limitations of this study is the relatively small sample size, particularly for local players, which may have reduced the statistical power of the findings. This limitation could have masked subtle differences in performance metrics and biomechanical variations between batting backlift techniques. Additionally, the study relied on video footage (2D) for international players, which, despite rigorous selection criteria, may not have provided the same level of precision in biomechanical measurements as in-person data collection. The use of publicly available footage also limited the ability to control for variations in camera angles, frame rates, image distortion and quality; potentially affecting the accuracy of backlift classifications. A further limitation involves the difference in data collection environments between the two groups. While local player data was collected under structured testing conditions, international data was sourced from match footage. Although this may introduce variability, strict inclusion criteria (e.g., right-arm bowlers, consistent camera positioning) were applied to maintain comparability across datasets. These two datasets served complementary roles. Local data offered precise biomechanical insights, while international footage provided real-match context and broader representation. An additional limitation was scheduling constraints related to local player availability and the cricket off-season. Furthermore, while this study provides valuable insights into female cricket biomechanics, it does not account for other influential factors such as bowler type, playing conditions or psychological aspects of batting technique. Future research should incorporate a larger and more diverse sample, controlled experimental settings and real-time motion capture to enhance the reliability and applicability of the findings.

## Conclusion

The findings of this study suggest that the LBBT was widely adopted by both local (67%) and international (62%) players, with no single technique dominating across all scoring areas. Performance metrics such as bat speed, impact speed and follow-through angles varied significantly among LBBT players, suggesting potential for higher peak performance. The study emphasises the importance of biomechanical efficiency and power generation, highlighting the need for tailored coaching strategies for female players. Practical implications include refining the LBBT, developing targeted drills, considering physiological differences and leveraging data-driven coaching for individualised feedback. Future longitudinal research studies should focus on three-dimensional (3D) motion capture (as well as markerless motion capture) on the LBBT, integrating real-time analytics and expanding sample sizes to include diverse player populations. This research study demonstrates the importance of gender-specific coaching and biomechanical optimisation among female cricket performance and female sports in general.

## Supplementary Information



## Figures and Tables

**Table 1:** Summary of the data captured for each group

Local players	International players
Video footage for each player	
Anthropometry *(height, weight, waist circumference, BMI, body fat percentage)*	Player career statistics *(matches, innings, not outs, runs, high score, career batting average)*
StanceBeam Smart Bat Data (*bat speed (km·h^−1^), impact speed (km·h^−1^), time to impact (milliseconds), downswing angle [degrees], follow-through angle [degrees], number of wagon wheels (on-side, off-side and straight); as well as the maximum bat speed (km·h^−1^) and maximum impact speed (km·h^−1^), vertical and horizontal shots)*	

**Table 2:** A comparison of straight and lateral batting backlift technique (BBT) in terms of anthropometric and performance metrics

Metric	BBT	n	Mean	SD	Mean rank

BMI	Straight	6	25.5	2.9	10.0
	Lateral	12	25.1	3.3	9.3

Waist circumference (cm)	Straight	6	73.6	4.5	8.8
	Lateral	12	75.6	6.0	9.8

Skinfolds (mm)	Straight	6	23.1	3.5	10.3
	Lateral	12	22.3	3.9	9.1

Bat Speed	Straight	6	35.8	6.5	7.9
	Lateral	12	37.8	6.9	10.3

Impact Speed (km·h^−1^)	Straight	6	34.0	5.5	9.3
	Lateral	12	36.4	6.9	9.6

Down-swing Angle (°)	Straight	6	126.5	20.4	8.4
	Lateral	12	132.5	21.1	10.0

Follow-through Angle (°)	Straight	6	45.2	15.3	9.2
	Lateral	12	46.4	15.1	9.7

Wagon Wheel – on-side	Straight	6	11.3	1.0	5.9
	Lateral	12	12.8	1.6	11.3

Wagon Wheel – off-side	Straight	6	1.5	1.5	11.8
	Lateral	12	0.6	0.9	8.3

Wagon Wheel - Straight	Straight	6	0.2	0.4	9.5
	Lateral	12	0.2	0.4	9.5

Max Bat Speed (km·h^−1^)	Straight	6	48.7	4.7	7.3
	Lateral	12	55.2	10.7	10.6

Max Impact Speed (km·h^−1)^	Straight	6	48.7	4.7	7.3
	Lateral	12	55.2	10.7	10.6

Max Power (W)	Straight	6	1210.3	178.4	7.9
	Lateral	12	1282.9	178.9	10.3

n, number of participants in each group; SD, standard deviation

**Table 3:** Mann-Whitney U Test of international players (IP) (LBBT versus SBBT group statistics)

Metric	BBT	n	Mean	SD	Mean rank

Matches	Straight	13	115	34	18.9
	Lateral	21	104	49	16.6

Innings	Straight	13	105	33	19.0
	Lateral	21	96	45	16.6

Not Outs	Straight	13	15	6	17.4
	Lateral	21	17	14	17.6

Runs	Straight	13	3448	1230	19.6
	Lateral	21	3097	1615	16.2

High Score	Straight	13	161	26	24.2
	Lateral	21	128	27	13.3

Average	Straight	13	38	5	18.0
	Lateral	21	39	8	17.2

n, number of participants in each group; SD, standard deviation; SBBT, straight batting backlift technique; LBBT, lateral batting backlift technique

**Table 4:** Cross tabulation of local players and international players

			BBT		Total

			Straight	Lateral	

**Groups**	**Local**	n	6	12	18
		%	33%	67%	100%

	**International**	n	13	21	34
		%	38%	62%	100%

**Total**		n	19	33	52
		%	37%	63%	100%

n, number of participants in each group
