# Supplementary material for: Does the backlift also matter in female cricket? A biomechanical investigation among local and international female cricket batters
Source: S Afr J Sports Med. 2025 Oct 15;37(1):v37i1a22315. doi: 10.17159/2078-516X/2025/v37i1a22315 (PMC12626433; doi:10.17159/2078-516X/2025/v37i1a22315)
Supplement: Supplementary file 1 [file 2078-516x-37-v37i1a22315-s001.pdf]

Does the backlift also matter in female cricket? A biomechanical investigation among local and international female cricket batters

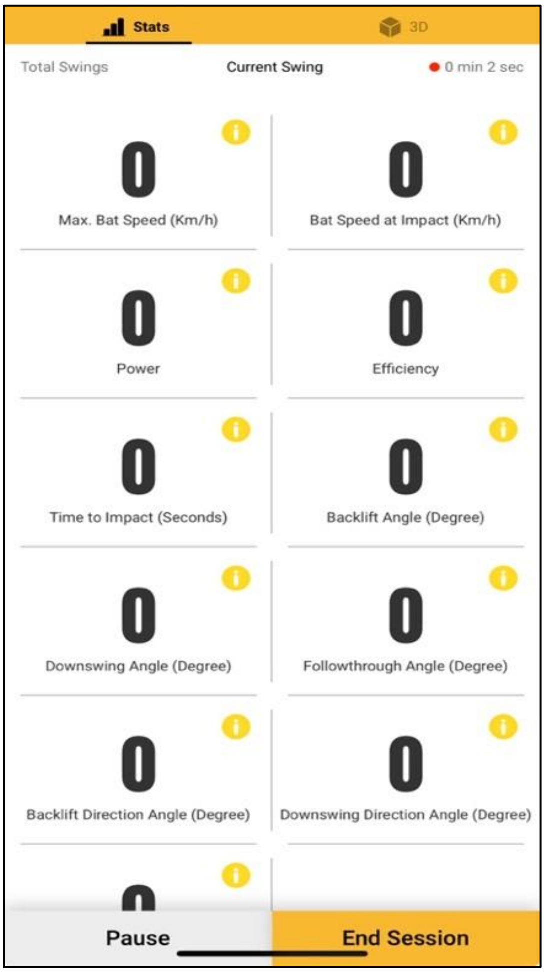

Supplementary Fig.1*. Screenshot of the StanceBeam current session page*

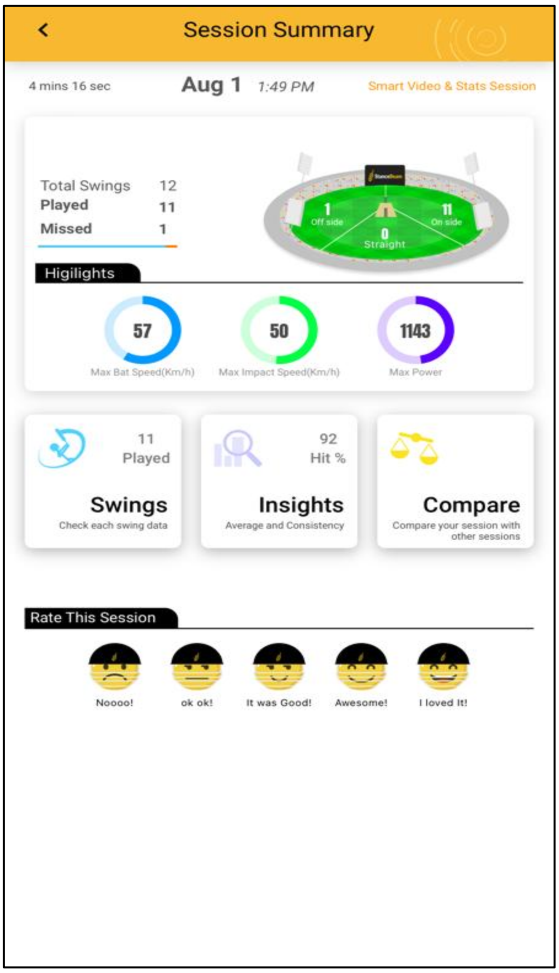

Supplementary Fig. 2*. Screenshot of the StanceBeam session summary page*

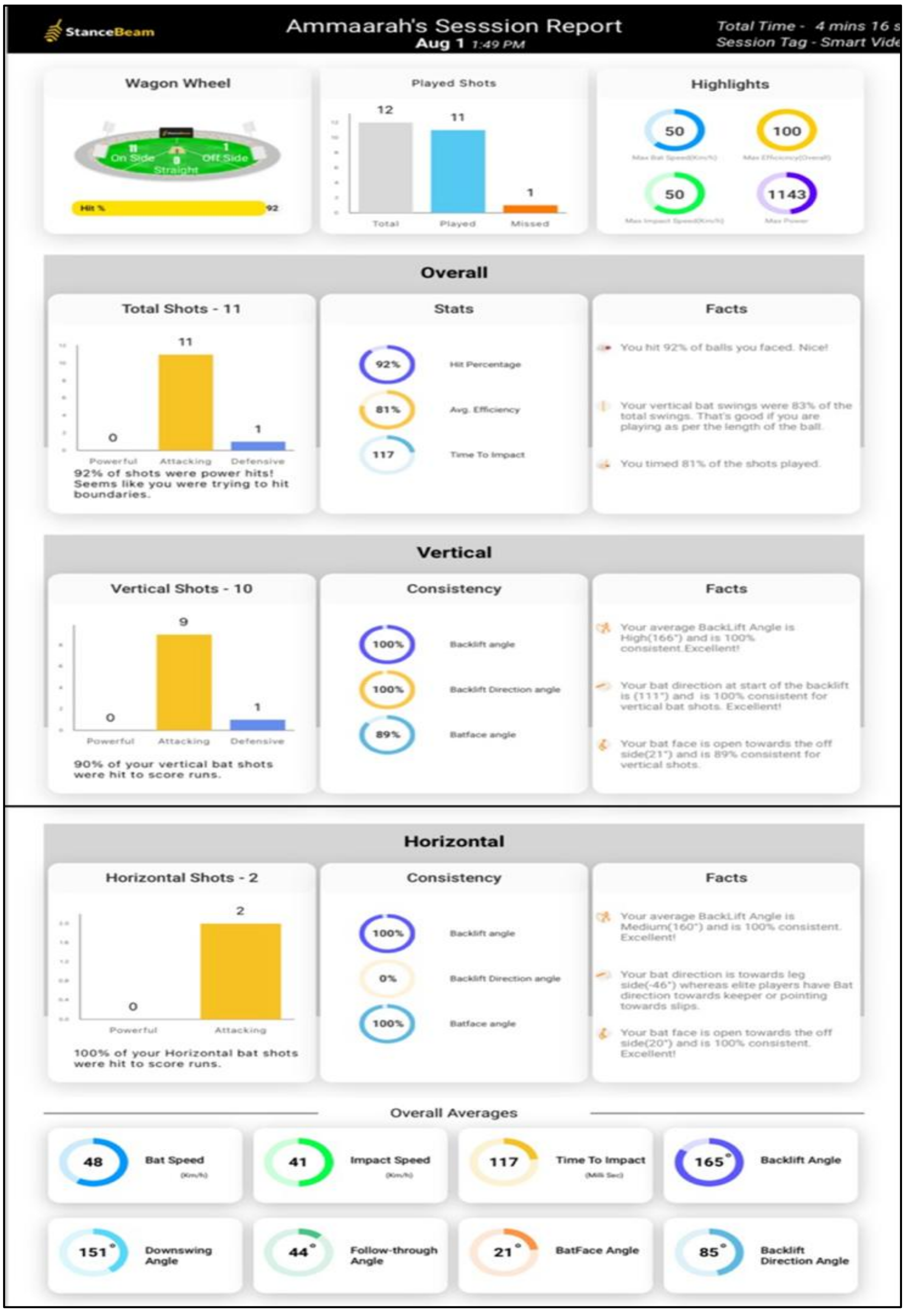

Supplementary Fig. 3. *Screenshot of the StanceBeam session report*

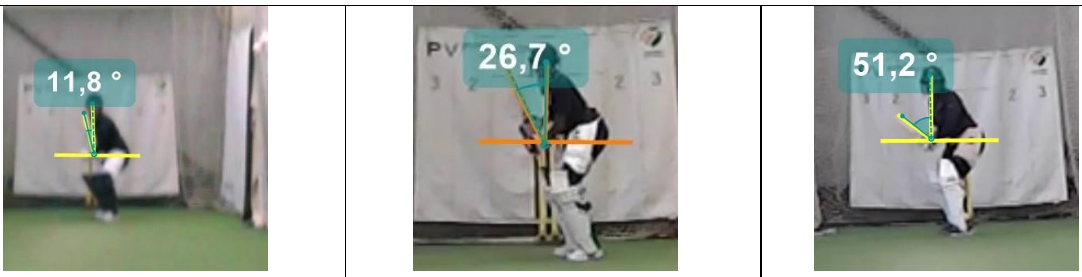

Classifier A	Classifier B	Classifier C

Supplementary Fig. 4. *Example illustrations of the BBT classifiers*

Supplementary Fig. 5. *Backlift analysis in relation to scoring areas of international players (video data of players were obtained from an online public source).*

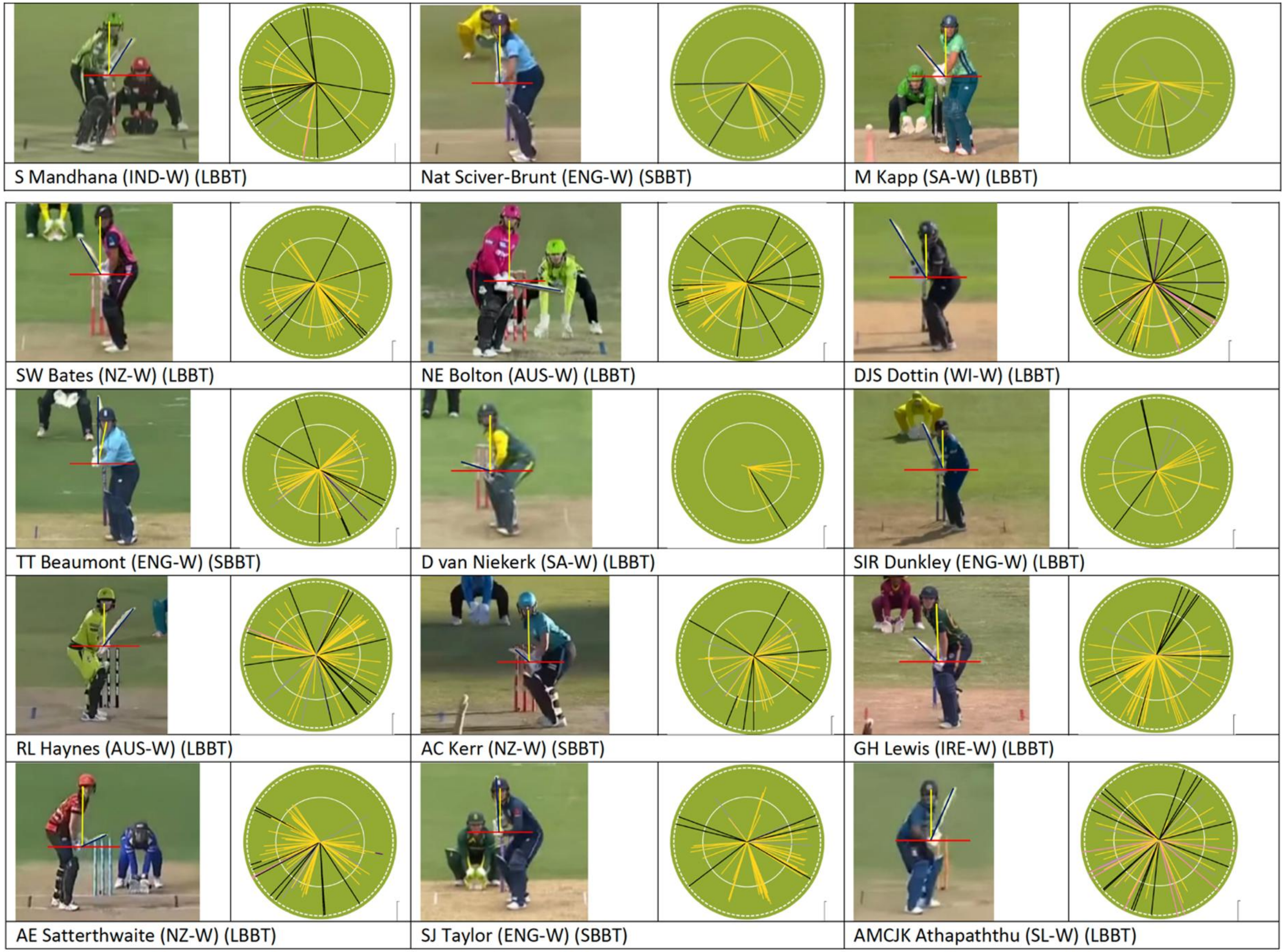

Continued: S*upplementary Fig. 5.* *Backlift analysis in relation to scoring areas of international players (video data of players were obtained from an online public source).*

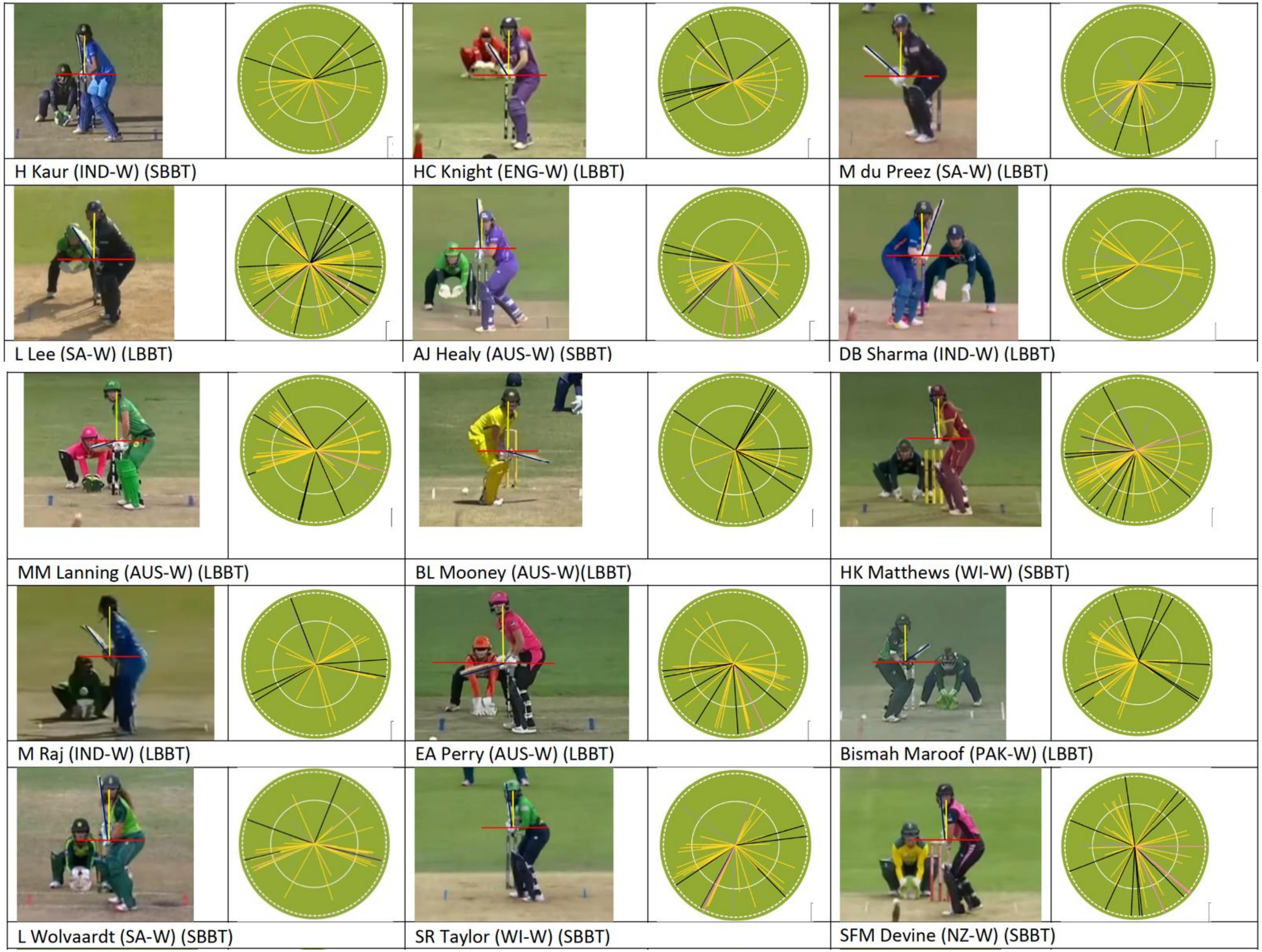

Supplementary Table 1. Correlations between bat speed, impact speed and follow-through angle among local players (n = 18)

Variable	BMI	WC	BF%	Bat Speed	Impact Speed	Down SA	Follow TA	WW1	WW2	WW3	Max Bat Speed	Max Impact Speed	Max Power	Vertical Shot	(VS) Backlift Height	(VS) Bat Direction	Horizontal Shot	(HS) Backlift Height	(HS) Bat Direction
**BMI**																			
**WC**	r=0.63<br>p=0.01																		
**BF%**	r=0.64<br>p=0.01	r=0.73<br>p=0.00																	
**Bat Speed**	r=0.06<br>p=0.11	r=0.26<br>p=0.30	r=0.27<br>p=0.28																
**Impact Speed**	r=0.20<br>p=0.44	r=0.26<br>p=0.30	r=0.34<br>p=0.16	r=0.12<br>p=0.00															
**Down SA**	r=-0.06<br>p=0.11	r=0.07<br>p=0.79	r=0.10<br>p=0.70	r=0.04<br>p=0.00	r=0.12<br>p=0.00														
**Follow TA**	r=0.23<br>p=0.36	r=0.28<br>p=0.26	r=0.39<br>p=0.11	r=0.11<br>p=0.00	r=0.77<br>p=0.00	r=0.60<br>p=0.01													
**WW1**	r=0.07<br>p=0.78	r=0.21<br>p=0.41	r=0.00<br>p=0.99	r=0.08<br>p=0.75	r=0.11<br>p=0.64	r=0.09<br>p=0.71	r=0.04<br>p=0.86												
**WW2**	r=0.41<br>p=0.10	r=0.29<br>p=0.24	r=0.44<br>p=0.07	r=-0.18<br>p=0.41	r=-0.08<br>p=0.76	r=-0.19<br>p=0.46	r=-0.24<br>p=0.35	r=-0.18<br>p=0.47											
**WW3**	r=-0.05<br>p=0.05	r=-0.27<br>p=0.27	r=-0.11<br>p=0.05	r=0.07<br>p=0.78	r=0.057<br>p=0.73	r=0.01<br>p=0.96	r=0.16<br>p=0.53	r=-0.34<br>p=0.16	r=-0.42<br>p=0.08										
**Max Bat Speed**	r=-0.03<br>p=0.92	r=0.13<br>p=0.62	r=0.10<br>p=0.70	r=0.41<br>p=0.04	r=0.53<br>p=0.03	r=0.44<br>p=0.07	r=0.32<br>p=0.19	r=0.52<br>p=0.03	r=0.16<br>p=0.52	r=-0.27<br>p=0.27									
**Max Impact Speed**	r=-0.03<br>p=0.92	r=0.13<br>p=0.62	r=0.10<br>p=0.71	r=0.41<br>p=0.04	r=0.53<br>p=0.03	r=0.44<br>p=0.07	r=0.32<br>p=0.19	r=0.52<br>p=0.03	r=0.16<br>p=0.52	r=-0.27<br>p=0.27	r=1.00<br>p=0.00								
**Max Power**	r=0.04<br>p=0.11	r=0.13<br>p=0.62	r=0.15<br>p=0.57	r=0.17<br>p=0.50	r=0.07<br>p=0.80	r=-0.07<br>p=0.78	r=0.19<br>p=0.45	r=0.10<br>p=0.61	r=0.20<br>p=0.42	r=-0.10<br>p=0.07	r=0.32<br>p=0.20	r=0.32<br>p=0.20							
**Vertical Shot**	r=-0.26<br>p=0.91	r=0.03<br>p=0.92	r=0.24<br>p=0.33	r=0.57<br>p=0.01	r=0.39<br>p=0.11	r=0.46<br>p=0.06	r=0.52<br>p=0.03	r=0.22<br>p=0.37	r=0.02<br>p=0.95	r=0.04<br>p=0.12	r=0.41<br>p=0.04	r=0.48<br>p=0.04	r=0.16<br>p=0.52						
**(VS) Backlift Height**	r=0.13<br>p=0.61	r=0.36<br>p=0.15	r=0.16<br>p=0.54	r=0.47<br>p=0.049	r=0.54<br>p=0.02	r=0.68<br>p=0.002	r=0.28<br>p=0.258	r=0.24<br>p=0.35	r=-0.02<br>p=0.93	r=-0.34<br>p=0.11	r=0.28<br>p=0.258	r=0.28<br>p=0.26	r=-0.16<br>p=0.53	r=-0.06<br>p=0.51					
**(VS) Bat Direction**	r=0.24<br>p=0.36	r=0.18<br>p=0.495	r=0.20<br>p=0.45	r=0.51<br>p=0.036	r=0.43<br>p=0.05	r=0.54<br>p=0.027	r=0.14<br>p=0.13	r=0.28<br>p=0.27	r=0.04<br>p=0.869	r=-0.54<br>p=0.03	r=0.38<br>p=0.14	r=0.38<br>p=0.14	r=0.07<br>p=0.79	r=0.21<br>p=0.42	r=0.54<br>p=0.03				
**Horizontal Shot**	r=0.12<br>p=0.65	r=0.11<br>p=0.406	r=-0.11<br>p=0.67	r=-0.62<br>p=0.006	r=-0.44<br>p=0.07	r=-0.54<br>p=0.01	r=-0.61<br>p=0.01	r=0.11<br>p=0.46	r=0.25<br>p=0.32	r=-0.18<br>p=0.41	r=-0.21<br>p=0.41	r=-0.21<br>p=0.41	r=0.11<br>p=0.67	r=-0.80<br>p=0.00	r=-0.067<br>p=0.71	r=-0.17<br>p=0.51			
**(HS) Backlift Height**	r=0.12<br>p=0.64	r=0.15<br>p=0.58	r=0.16<br>p=0.55	r=-0.01<br>p=0.76	r=0.00<br>p=0.10	r=0.21<br>p=0.43	r=-0.30<br>p=0.25	r=0.26<br>p=0.341	r=0.25<br>p=0.29	r=-0.19<br>p=0.49	r=0.11<br>p=0.49	r=0.18<br>p=0.50	r=-0.27<br>p=0.31	r=0.15<br>p=0.58	r=0.30<br>p=0.26	r=-0.16<br>p=0.57	r=0.10<br>p=0.71		
**(HS) Bat Direction**	r=-0.24<br>p=0.44	r=-0.07<br>p=0.11	r=-0.31<br>p=0.30	r=-0.25<br>p=0.40	r=-0.21<br>p=0.49	r=-0.18<br>p=0.558	r=-0.211<br>p=0.35	r=-0.01<br>p=0.97	r=-0.30<br>p=0.33	r=0.00<br>p=1.00	r=-0.27<br>p=0.37	r=-0.27<br>p=0.37	r=-0.21<br>p=0.50	r=-0.48<br>p=0.10	r=0.27<br>p=0.37	r=0.02<br>p=0.96	r=0.19<br>p=0.52	r=-0.29<br>p=0.34	

BMI, body mass index; WC, waist circumference; BF%, body fat percentage; Down SA, downswing angle; Follow TA, follow-through angle; WW, wagon wheel; VS, vertical shot; HS, horizontal shot
